# Supplementary material for: Evaluation of Bayesian Linear Regression models for gene set prioritization in complex diseases
Source: PLoS Genet. 2024 Nov 4;20(11):e1011463. doi: 10.1371/journal.pgen.1011463 (PMC11563439; doi:10.1371/journal.pgen.1011463)
Supplement: S1 Text — (PDF) [file pgen.1011463.s001.pdf]

UKB data for simulations

UKB genotyped and imputed data were used for simulations analysis of the UKB phenotypes. In our study, we had information about 488,377 participants. To obtain a genetic homogeneous study population we restricted our analyses to unrelated British Caucasians and excluded individuals with more than 5,000 missing markers or individuals with autosomal aneuploidy. Remaining (n=335,532) White British unrelated individuals (WBU) were used for analyses. Then, we excluded markers with minor allele frequency < 0.01, call rate < 0.95 and the markers deviating from Hardy-Weinberg equilibrium (P-value $< 1 \times 10^{-12}$). We excluded markers located within the major histocompatibility complex (MHC), having ambiguous allele (i.e., GC or AT), were multi-allelic or an indel (1). This resulted in a total of 533,679 single nucleotide polymorphism (SNP) markers in the simulated data.

Genetic architectures for simulations

To simulate genetic architectures from low to high polygenicity, we simulated quantitative phenotypes with heritability (h^2_{SNP}) of 30% and 10%, with two different proportions of causal SNPs (π), 0.1% and 1%, chosen randomly from the genome.

We generated two different types of genetic architectures under a multiple regression model. In the first genetic architecture (GA_1), causal SNPs (m_C) effects were sampled from the same normal distribution:

$$y_i = \sum_{j=1}^{m_C} w_{ij}\, b_j + e_i,$$

where y_i is the phenotype for i'th individual, b_j is the estimate of the j'th SNP effect (normally distributed with mean of 0 and variance given by σ_g^2/m_C). We assumed variance of a phenotype

to be 1 such that σ_g^2 is equal to h_{snp}^2. w_{ij} represents the j'th centered and scaled genotype of the i'th individual:

$$w_{ij} = \frac{x_{ij} - 2p_j}{\sqrt{2p_j(1 - p_j)}}$$

where, x_{ij} is the effect allele count for i'th individual at the j'th SNP, p_j is the allele frequency of the j'th SNP. e_i is the residual that has a normal distribution with mean=0 and variance= $\sigma_g^2(1 / (h_{snp}^2) - 1)$. Residual variance was scaled in a way so that h_{snp}^2 remained 30% (or 10%).

In the second genetic architecture scenario (GA_2), the effects of causal SNPs are sampled from a mixture of normal distributions.

$$y_i = \sum_{j=1}^{m_{C_1}} w_{ij}\, b_j + \sum_{k=1}^{m_{C_2}} w_{ik}\, b_k + \sum_{l=1}^{m_{C_3}} w_{il}\, b_l + e_i,$$

where, b_j , b_k , and b_l are the effect of causal SNPs sampled from normal distribution with mean=0 and variance = $(0.6\sigma_g^2)/(0.93m_C)$, $(0.2\sigma_g^2)/(0.05m_C)$, and $(0.2\sigma_g^2)/(0.02m_C)$ respectively. In this genetic model, the three normal distributions were designed such that 93% of the causal SNPs would have small effect sizes and the remaining 5% and 2% of the causal SNPs would have moderate and large effect sizes respectively. This genetic architecture was designed in a similar way as designed in the study by (2).

All the other parameters in GA_2 are created in a similar way as for the GA_1.

We created ten replicates for each simulation scenario. The total sample of 335,532 were divided into ten replicates. Each replicate contained 80% of the randomly sampled data from the total samples.

For the quantitative phenotypes, a total of eight different simulation scenarios were applied: two values of h_{snp}^2, two different proportions of causal SNPs π and two different genetic architecture scenarios.

To simulate binary phenotypes, in addition to the parameters: h_{SNP}^2, π and genetic architectures, we introduced another parameter “sample disease prevalence” (PV). Two different PV of 5% and 15% were used in our study. We simulated binary phenotypes from quantitative phenotypes. To simulate a binary phenotype, for example with PV 5%, we chose top 5% of individuals with highest simulated quantitative values as cases and the remaining as controls for the total sample in a replicate. Each scenario of a quantitative phenotype gave rise to two different scenarios for binary phenotype. In total we designed 16 different simulation scenarios for the binary phenotypes: two values of h_{snp}^2, two different proportions of causal SNPs π, two different genetic architecture scenarios, and two prevalence PV. Different scenarios for the quantitative and the binary phenotypes are presented in detail in Table 1. The flowchart of design of the simulations is presented in S1 Fig.

Simulation of gene sets

We created a series of synthetic gene sets. These gene sets were constructed based on a predefined causal marker list derived from the different simulation scenarios described above (S2 Fig) . Initially, we categorized genes into two groups: causal genes, which contained causal markers, and non-causal genes, which do not contain the causal markers. To control the size and enrichment of causal genes within these gene sets, we employed two key parameters: the total number of genes in each gene set (referred to as the gene set size), ranging from 10 to 200 genes, and the number of causal genes (n) selected from causal genes (3). We explored different values for number of causal genes, including 0, 5, 10, 25, 50, 100, and 200. To reduce sampling bias, we conducted ten replicates for each gene set configuration. In total, we generated 21

distinct gene set configurations for each simulation scenario. Notably, configurations without any causal genes were used to assess the number of false positive in the evaluation metrics described below.

Run Time Comparison

We have evaluated the computational efficiency of MAGMA and BLR in terms of run time performance for real trait analysis. The mean run time for MAGMA across all single-trait analyses was approximately 0.0888 hours. In contrast, BLR demonstrated a significantly faster mean run time for single-trait analyses, averaging around 0.0392 hours.

For multi-trait analyses, we expanded our assessment to cover all possible combinations of 2 to 9 traits. The mean run times for these multi-trait analyses are presented in S5 Fig.

1. Andries TM, Hilde de K, Sven S, Florence V, Emmanuel C, Cynthia M-C, et al. A tutorial on conducting genome-wide association studies: Quality control and statistical analysis. International Journal of Methods in Psychiatric Research. 2018;27:1-10. doi: 10.1002/mpr.1608. PubMed PMID: Marees2018.
2. Lloyd-Jones LR, Zeng J, Sidorenko J, Yengo L, Moser G, Kemper KE, et al. Improved polygenic prediction by Bayesian multiple regression on summary statistics. Nat Commun.

2019;10(1):5086. Epub 2019/11/11. doi: 10.1038/s41467-019-12653-0. PubMed PMID: 31704910; PubMed Central PMCID: PMC6841727.
3. Rohde PD, Demontis D, Cuyabano BCD, Group TGMfS, Børglum AD, Sørensen P. Covariance Association Test (CVAT) Identifies Genetic Markers Associated with Schizophrenia in Functionally Associated Biological Processes. Genetics. 2016;203(4):1901-13. doi: 10.1534/genetics.116.189498.
