# Supplementary figures and images for: Evaluation of Bayesian Linear Regression models for gene set prioritization in complex diseases
Source: PLoS Genet. 2024 Nov 4;20(11):e1011463. doi: 10.1371/journal.pgen.1011463 (PMC11563439; doi:10.1371/journal.pgen.1011463)

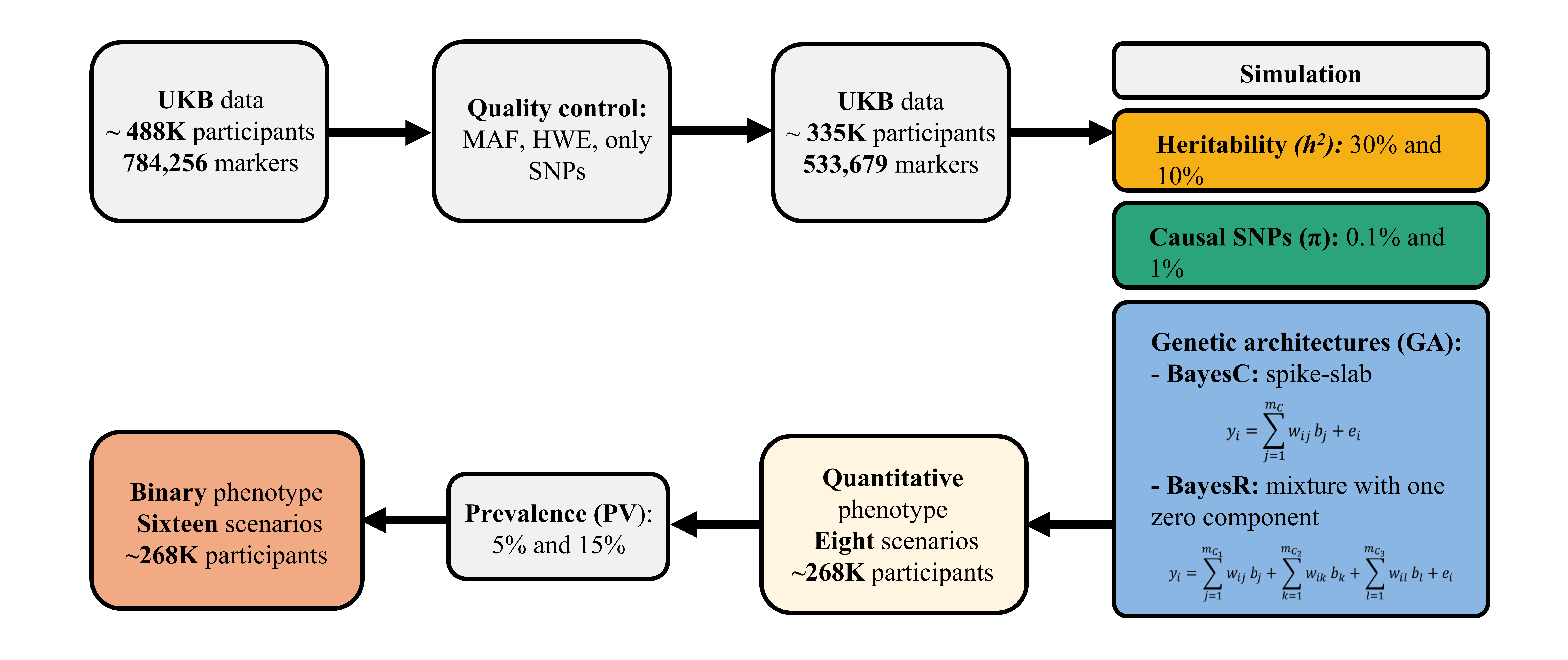

Supplement: S1 Fig — This figure outlines the process used to simulate quantitative and binary traits from UKB chip genotypes, including quality control measures and the generation of different simulation scenarios based on heritability, the proportion of causal variants, and disease prevalence. (TIF) [file pgen.1011463.s002.tif]

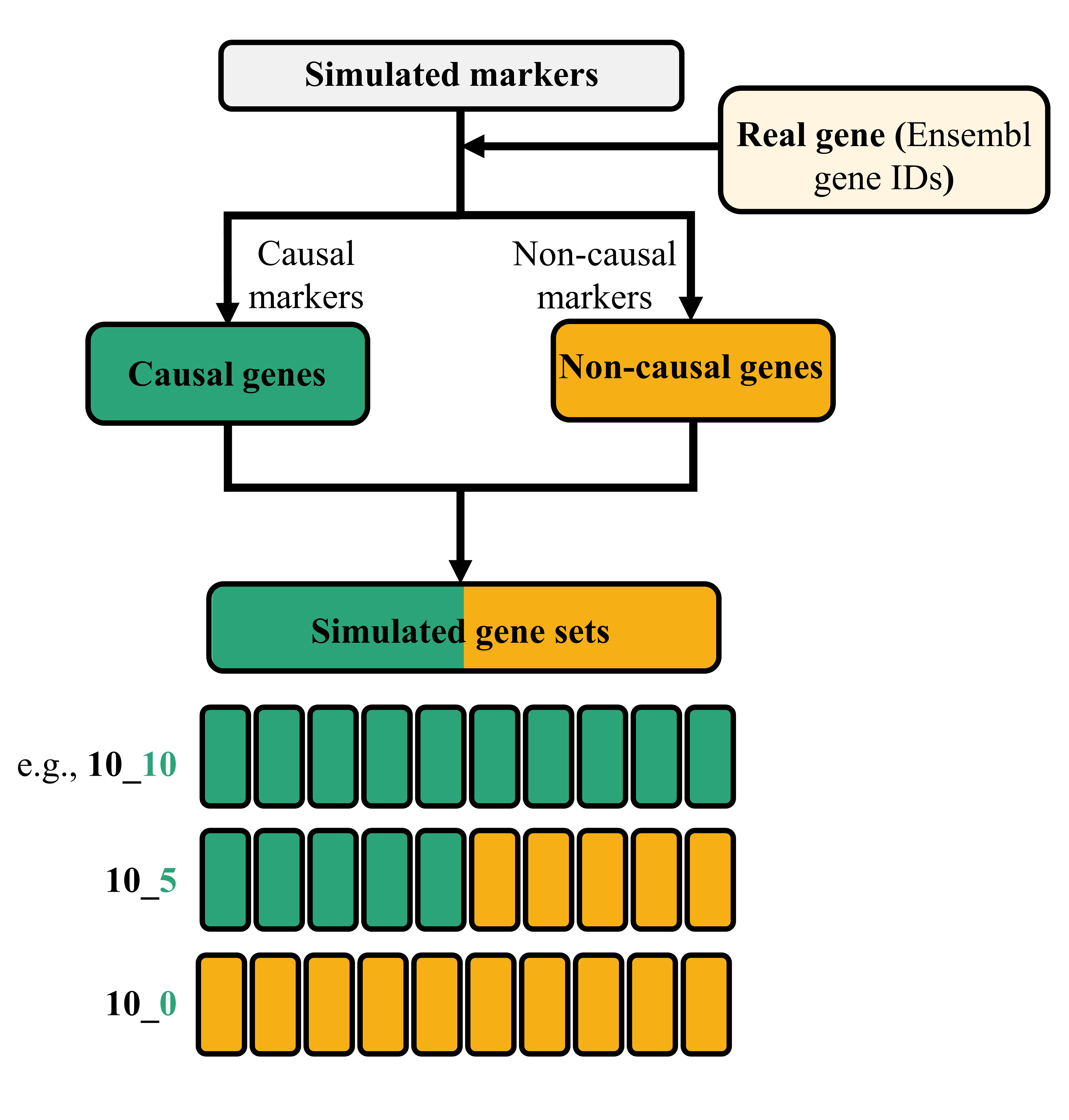

Supplement: S2 Fig — This figure describes the process used to create synthetic genes and gene sets based on simulated SNPs and their genomic locations. (TIF) [file pgen.1011463.s003.tif]

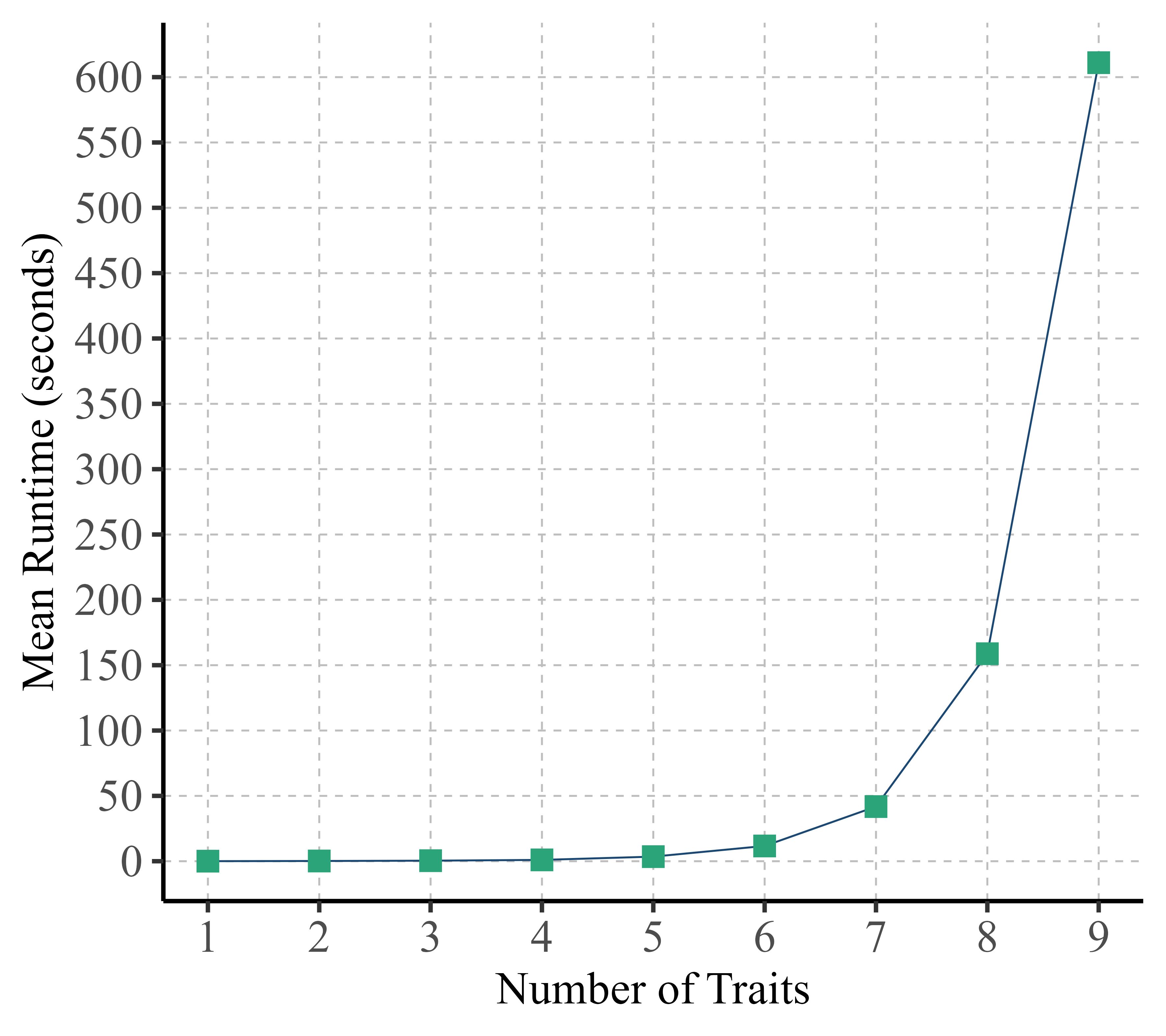

Supplement: S5 Fig — The mean run times for the BLR model in multi-trait analyses. All possible combinations of 2 to 9 traits were analyzed, and the mean run times are shown. (TIFF) [file pgen.1011463.s006.tiff]
